# Cervical musculoskeletal, physical and psychological factors associated with ongoing dizziness in patients with whiplash associated disorder, 12 months after undertaking a neck specific or general exercise intervention

**DOI:** 10.1186/s12891-022-05642-w

**Published:** 2022-07-18

**Authors:** Julia Treleaven, Gunnel Peterson, Maria Landén Ludvigsson, Anneli Peolsson

**Affiliations:** 1 The Division of Physiotherapy, CCRE-Spine, School of Health and Rehabilitation Sciences, The University of Queensland, Brisbane, Australia; 2 Department of Health, Medicine and Caring Sciences, Unit of Physiotherapy, Linköping University, Linköping, Sweden; 3 Centre for Clinical Research Sörmland, Uppsala University, Uppsala, Sweden; 4 Rehab Väst, County Council of Östergötland, Department of Rehabilitation and Department of Health, Medicine and Caring Sciences, Linköping University, Motala, Sweden; 5 Occupational and Environmental Medicine Centre, Department of Health, Medicine and Caring Sciences, Unit of Clinical Medicine, Linköping University, Linköping, Sweden

**Keywords:** Dizziness, Balance, Whiplash injury, Exercise therapy, Predictive factors

## Abstract

**Background:**

Exercise in the management of persistent whiplash often doesn’t specifically address dizziness. This study aimed to determine cervical musculoskeletal and sensorimotor measures, quality of life and psychological factors associated with the presence of dizziness in individuals with persistent whiplash 12 months post exercise intervention commencement.

**Methods:**

A retrospective cross sectional review of questionnaires on dizziness, physical and psychological disability, quality of life and physical measures prospectively collected from 172 individuals during a randomised controlled trial. Associations between dizziness at 12 months post intervention and possible predictors was analysed with simple and multiple logistic regression models.

**Results:**

Sixty-three % reported dizziness with a mean University of California Los Angeles dizziness score of 9 (SD 5) and dizziness intensity during activity of 26 mm (SD 24). They had poorer performance on sharpened Rhomberg, Neck muscle endurance (NME), and range of motion, elevated scores on pain, Neck disability index (NDI) and psychological and quality of life measures compared to those without dizziness. Less improvement in NDI and NME flexion from baseline to 12 months post exercise commencement, along with some baseline covariates were related to persistent dizziness and explained 50% of the variance.

**Conclusion:**

Dizziness following exercise at 12 months post follow-up was associated with lack of improvement in NDI and NME flexion suggesting a cervicogenic role. Alternatively, the presence of dizziness may inhibit exercise response. Additional causes or contributing factors of dizziness should be investigated in those with persistent whiplash to improve quality of life.

## Background

It is estimated that up to 50% of persons sustaining neck trauma as a result of a motor vehicle collision will go on to have persistent problems [1] with multifactorial causes including biological, psychological and social factors [2, 3]. These patients present challenges to all professionals involved and present with a variety of symptoms. Understanding the relationships between these signs and symptoms is important to direct rehabilitation.

After pain, dizziness and unsteadiness is a frequent complaint, with up to 70% of those with persistent whiplash associated disorder (WAD) reporting these complaints [4]. These symptoms are thought to reflect abnormal cervical afferent to the sensorimotor control system in most patients and have been associated with objective deficits in head and eye movement control and postural stability relevant to a cervical cause [4–6]. A variety of causes of abnormal cervical afferent input following a whiplash injury have been highlighted in the literature including functional impairment of muscles, such as altered neuromotor control or increased fatigability [7]. In addition, the effects of pain at many levels of the nervous system can change muscle spindle sensitivity and alter the cortical representation and modulation of cervical afferent input [8] Psychosocial stresses may also influence muscle spindle activity via activation of the sympathetic nervous system [9].

To date there is modest evidence for the effect of exercise in the management of WAD on pain and disability [10]. These treatments address some of the causes of altered cervical afferent input but do not specifically address factors associated with sensorimotor control such as dizziness, proprioception and balance. Previous studies have determined the effect of a specific neck, a combined specific neck and behavioural approach or a general exercise program on pain and disability [11] in persistent WAD and then specifically considered the effects of this program on symptoms of dizziness and deficits in sensorimotor control in those reporting dizziness and postural instability [12]. The later study found that although between and within group comparisons suggested that those performing the neck specific exercise had significant advantages in improving measures of dizziness and proprioception compared with the general physical exercise group, many still complained of dizziness and balance impairment at the 12-month follow-up [12].

To assist direction for management of dizziness in persistent WAD it will be important to understand factors associated with ongoing complaints of dizziness. Thus the aim of this study was to first compare cervical related physical and psychological factors in individuals with and without dizziness, 12 months after a neck specific or general exercise intervention and secondly to determine the combination of these factors to best predict those reporting ongoing dizziness. It was hypothesised that a combination of physical and psychological factors would predict those reporting ongoing dizziness.

## Methods

This was a retrospective review of prospectively gathered data collected during a randomized controlled trial (RCT) (*n* = 216, mean age of 40 (SD 11), 65% women). A detailed description of the RCT study design and the 12 week intervention can be found in [11, 12]. The interventions included A) Physiotherapist-guided neck specific exercise B) Physiotherapist-guided neck specific exercise, with a behavioural approach and C) Prescription of general Physical Activity.

Briefly, patients with a WAD diagnosis, at least 6 months but no more than 3 years after a motor vehicle collision, who fulfilled the eligibility criteria attended a physical examination to ensure eligibility. To be included in the original study participants had to be a WAD II or III [13] and have continuing pain (> 20 mm on 100 mm Visual Analogue Scale (VAS) [14] and/or > 20% on Neck Disability Index (NDI), 0–100%) [15]. Subjects were excluded if they had known or suspected serious physical pathology, earlier neck trauma, surgery or neck pain with persistent injury, signs of traumatic vestibular or brain injury at the time of WAD, generalized or more dominant pain elsewhere in the body, diseases or other injuries that might prevent full participation in the study, diagnosed severe psychiatric disorder or known drug abuse.

All measurements were conducted at baseline and 12 months post commencement of the intervention. Questionnaires covered aspects relating to dizziness and pain intensity and disability, psychological (catastrophysing, kinesiophobia, selfefficacy, depression and anxiety) and health related quality of life measures. Clinical neck related measurements included cervical joint position sense, cervical range of motion (ROM) and neck flexor and extensor muscle endurance measures (NME). Measures of static (eyes closed rhomberg) and dynamic (figure of eight walk) balance were also considered. Information pertaining to all measurements, references regarding their psychometric properties and their abbreviations are included in Table 1. These were performed in a standardised way by well-trained investigators. Demographic data was also obtained.

**Table 1:** Questionnaires and physical measures performed at baseline and at 12 months post intervention

Questionnaires			Unit/Range	Reference
*Dizziness and unsteadiness*	Do you have dizziness	YES /NO	N/A	
	Dizziness intensity at rest Dizziness intensity during activity Unsteadiness intensity	VAS VAS VAS	0-100 mm	**[**14**]**
	University of California Los Angeles Dizziness Questionnaire (UCLA)	5 item questionnaire severity, frequency and fear of dizziness and its effect on quality of life and activities of daily living	5–25	**[**16**]**
*Pain and disability*	Neck pain intensity now Neck pain intensity worst	VAS VAS	0-100 mm	**[**14**]**
	Neck Disability Index (NDI)	10 item neck specific function	0–100%	**[**15**]**
	Pain Disability Index	Specific and general disability related to chronic pain	0–70	**[**17**]**
*Psychological and quality of life*	The Pain Catastrophizing Scale (PCS).	Score above 30 thought to be indicative of catastrophic thinking	0–52	**[**18**]**
	The Self-Efficacy Scale (SES).	Higher scores indicate greater self-efficacy	0–200	**[**19**]**
	The Tampa Scale of Kinesiophobia (TSK).	Higher scores indicate higher fear of movement	11–44	**[**19**]**
	Hospital Anxiety and Depression anxiety subscale (HAD-A) Hospital Anxiety and Depression depression subscales (HAD-D)	Scores > 10 suggested to indicate probable anxiety Scores > 10 suggested to indicate probable depression	0–21 0–21	**[**20**]**
	Health related quality of life with EuroQuol (EQ-5D-3L).	An index of 1 indicating highest quality of life	−.594–1.00	**[**21**]**
**Physical measures**				
*Sensorimotor*	Head relocation accuracy (HRA)	Ability to reproduce the neutral head position with the eyes closed was measured using the CROM. Average HRA both directions.	Degrees	**[**22**]**
	Static clinical balance test	The time (up to 30 seconds) able to maintain sharpened Romberg’s with eyes closed, non-dominant foot in front	Seconds	**[**23**]**
	Dynamic clinical balance test:	Walking in a figure 8-incorrect steps	Number	**[**24**]**
*Range of motion*	Cervical Range of motion (ROM)	Active ROM in all 3 planes measured using the CROM	Degrees	**[**25**]**
*Muscle endurance*	Cervical extensors Cervical flexors	Holding time in prone position Holding time in supine position	Seconds Seconds	**[**26**]**

References in bold support that the measurements have acceptable measurement properties

### Data management and statistical analysis

**Fig. 1:**
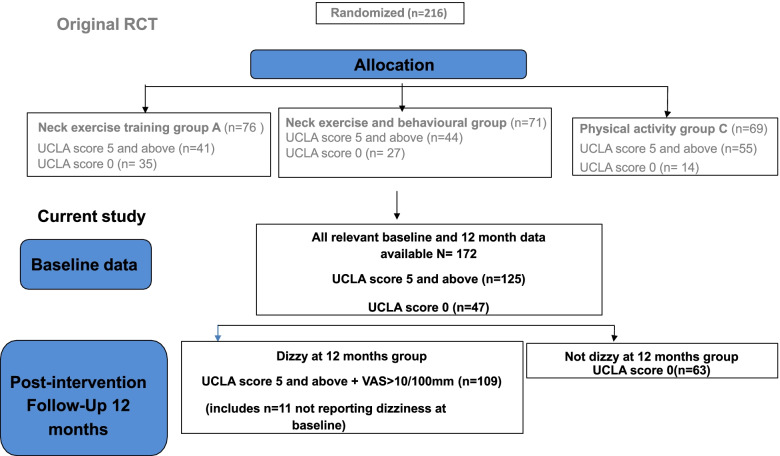
Dizziness at 12 months follow-up and baseline, participants in the study (*n* = 172)

For the purposes of this study only data from participants who had completed all relevant measures at both baseline and at the 12-month follow-up as well as answered a yes/ no question on dizziness and questions regarding dizziness and unsteadiness intensity at the 12 month mark were included. Data from eligible participants for this study was then pooled into two groups, no dizziness and dizziness, according to whether they complained of dizziness and unsteadiness at the 12-month follow-up or not, regardless of the intervention group. To be included in the dizziness at 12-months group, participants had to report dizziness, complete the University of California Los Angeles Dizziness (UCLA) questionnaire, and also rate more than 10 mm on either of the dizziness intensity or unsteadiness VAS (Fig. 1).

Between group differences for age at baseline, and dizziness intensity and unsteadiness, pain and disability, psychological and quality of life measures and physical measures at baseline, 12 months, and change between baseline and 12 months, were analysed with independent sample t-tests. All measures were presented as mean and standard deviation (SD) in Table 2. Cohen’s *d* was calculated as an effect size measure in measures where the *p*-value <.05 was seen in the between group difference in change at 12 months.

**Table 2:** Descriptive statistics on all measures at baseline, at 12 months, and change between baseline and 12 months

		Baseline			12 months			Change at 12 months					
		No dizziness^a^	Dizziness^a^	Between group difference	No dizziness^a^	Dizziness^a^	Between group difference	No dizziness^a^		Dizziness^a^		Between group difference	
Type of measures	Variables	Mean (SD)	Mean (SD)	*p*-value	Mean (SD)	Mean (SD)	*p*-value	n	Mean (SD)	n	Mean (SD)	*p*-value	Effect size
	Age	39.2 (10.9)	42.5 (11.5)	.072									
Dizziness/ unsteadiness	VAS dizziness at rest (0–100 mm)	2.4 (5.5)	17.9 (22.5)	<.001		11.6 (16.9)				108	6.4 (21.9)		
	VAS dizziness during activity (0–100 mm)	10.0 (18.5)	29.4 (27.3)	<.001		26.4 (24.4)				107	2.4 (27.2)		
	VAS disturbed balance (0–100 mm)	7.1 (13.3)	23.6 (25.1)	<.001		24.7 (26.3)				106	−1.6 (25.3)		
Sensorimotor physical	Sharpened Romberg (seconds)	16.3 (10.2)	12.3 (9.6)	.010	19.8 (10.0)	15.0 (10.7)	.005	59	3.8 (8.9)	105	2.6 (9.4)	.433	
	Figure of eight (no. of steps)	2.8 (4.0)	3.9 (4.9)	.147	1.1 (1.9)	1.5 (2.7)	.361	57	1.7 (3.3)	99	2.3 (3.4)	.313	
	HRA mean left + right (mm)	5.7 (4.1)	7.5 (6.5)	.023	4.4 (4.0)	4.9 (5.2)	.533	57	1.2 (5.3)	103	2.7 (7.0)	.164	
Pain and disability	VAS neck pain, now (0–100 mm)	33.8 (24.3)	45.8 (24.4)	.002	17.6 (23.6)	38.1 (26.3)	<.001	62	16.6 (27.2)	108	8.1 (22.8)	.031	.348
	Neck Disability Index (0–100%)	25.6 (10.8)	36.1 (13.0)	<.001	17.5 (13.7)	34.4 (16.8)	<.001	61	8.9 (13.1)	107	2.1 (13.3)	.001	.518
	Pain Disability Index (0–70)	14.3 (10.8)	23.6 (13.9)	<.001	8.7 (8.8)	22.1 (15.9)	<.001	62	5.8 (11.8)	108	1.7 (12.9)	.040	.330
Psychological	Pain Catastrophizing Scale (0–52)	16.0 (9.2)	18.5 (11.2)	.147	10.6 (9.4)	16.4 (12.4)	.002	60	4.7 (8.1)	106	2.0 (9.7)	.067	
	HAD Anxiety (0–21)	5.5 (3.5)	7.0 (4.4)	.023	4.4 (3.4)	6.5 (4.6)	.001	60	1.1 (3.2)	107	.5 (3.4)	.302	
	HAD Depression (0–21)	2.9 (3.1)	5.7 (4.4)	<.001	2.6 (2.9)	5.7 (4.6)	<.001	59	.3 (2.3)	107	.0 (3.8)	.599	
	Self-Efficacy Scale (0–200)	166.5 (31.4)	143.0 (34.5)	<.001	172.4 (30.3)	145.2 (42.3)	<.001	58	7.5 (30.9)	106	2.8 (33.8)	.375	
	Tampa Scale of Kinesiophobia (11–44)	20.3 (5.2)	22.8 (6.1)	.007	17.4 (5.2)	21.4 (7.1)	<.001	59	2.6 (4.6)	106	1.4 (6.1)	.196	
Musculoskeletal physical	NME, flexion (seconds)	43.0 (43.3)	33.3 (48.0)	.190	57.0 (50.6)	34.9 (36.4)	.005	56	17.2 (41.7)	103	.7 (36.2)	.010	.433
	NME, extension (seconds)	129.4 (143.0)	78.7 (110.7)	.018	195.7 (198.3)	119.9 (235.7)	.045	54	84.7 (186.0)	100	39.3 (202.8)	.174	
	ROM, flexion (degrees)	49.0 (15.4)	41.7 (14.3)	.002	52.8 (14.3)	46.7 (13.6)	.007	59	3.4 (13.7)	106	4.9 (12.3)	.478	
	ROM, extension (degrees)	57.1 (15.5)	50.4 (17.0)	.011	58.8 (15.1)	50.8 (18.3)	.003	59	2.1 (12.0)	106	.4 (13.1)	.420	
	ROM, flexion + extension (degrees)	106.0 (23.0)	92.1 (26.2)	.001	111.6 (23.9)	97.4 (28.2)	.001	59	5.5 (18.1)	106	5.3 (20.6)	.953	
	ROM, lateral flexion right + left (degrees)	69.8 (15.4)	63.5 (18.1)	.020	72.3 (16.2)	64.4 (18.5)	.007	59	2.3 (7.2)	106	1.0 (9.0)	.346	
	ROM, rotation right + left (degrees)	114.6 (22.3)	108.4 (28.9)	.115	124.9 (21.8)	111.2 (27.5)	.001	59	10.7 (16.6)	106	3.1 (22.5)	.014	.403
Quality of life	EQ-5D Index (−.594–1.000)	.698 (.180)	.581 (.273)	.001	.767 (.211)	.611 (.285)	<.001	60	.067 (.226)	105	.027 (.267)	.335	
	EQ-VAS (0–100 mm)	70.3 (14.6)	58.9 (18.4)	<.001	77.2 (16.7)	62.9 (21.4)	<.001	60	7.2 (16.8)	106	3.9 (23.8)	.310	

^a^Dizziness at 12 months follow-up is based on the yes/no question on dizziness and/or VAS > 10 mm in dizziness intensity/unsteadiness

*VAS* visual analogue scale; *HRA* head relocation accuracy; *NME* Neck muscle endurance; *ROM* range of motion, *HAD-A* HAD-D hospital anxiety and depression scales, *EQ-5D* EQ-VAS- EuroQuol quality of life index

Simple and multiple logistic regression models were used to analyse the association between the binary dependent variable of dizziness at 12 months and possible predictors, measured as change between baseline and 12 months follow-up. Both crude and adjusted odds ratios (OR) with 95% confidence intervals (CI) and Nagelkerke pseudo R^2^ were explored. Baseline measures, gender, age, WAD-level, intervention (neck specific exercise including behavioural OR physical activity), dizziness, and each predictor were entered as covariates in the adjusted models. Predictors with a *p*-value <.20 in the simple logistic regression models were entered in the multiple model, using backward stepwise procedure. There was no multicollinearity among the predictors, the variance inflation factor varied between 1.1 and 3.1, which can be interpreted as low to moderate correlation. Level of significance was set at *p* < .05. The IBM SPSS statistical program 25.0 was used for all calculations.

## Results

In total data from 172 of the original 216 participants were eligible for inclusion in this study. The mean age was 41 years (SD 11), and included 110 women, 97 were classified as WAD grade 2. While 125 participants complained of dizziness and 47 did not at baseline, at the 12-month assessment,109 participants reported dizziness and 63 did not. (Fig. 1).

Table 2 depicts all data comparing participants with and without dizziness at the 12-month follow-up. The group with dizziness had higher levels of: neck pain (VAS), disability- NDI, pain disability index (PDI), pain catastophysing scale (PCS), hospital anxiety and depression scales (HAD-A, HAD-D), tampa scale of kinesiophobia (TSK), poorer self efficacy scale (SES) and EuroQuol quality of life (EQ-VAS), poorer neck muscle endurance (NME) and static balance and less total ROM. According to HAD-A or HAD-D score s > 10, six (10%) participants showed probable anxiety and two (3%) probable depression in the non-dizzy group, while 26 (24%) participants showed probable anxiety and 18 (17%) probable depression in the dizzy group.

Table 3 depicts the results of the simple crude and adjusted logistic regression models on dizziness at the 12-month follow-up. Measures of change at 12 months in, VAS neck pain right now, NDI, PDI, NME flexors, and EQ-VAS showed significant association with dizziness at 12 months, based on the adjusted models.

**Table 3:** Simple crude and adjusted logistic regression models on dizziness at 12 months for relevant measures

Type of measures	Measures of change at 12 months	n	Crude					Adjusted^a^				
			B	Odds Ratio	95% CI for Odds Ratio	*p*-value	Pseudo R^2^	B	Odds Ratio	95% CI for Odds Ratio	*p*-value	Pseudo R^2^
Pain and disability	VAS neck pain, now (0–100 mm)	170	−.014	.986	.973–.999	.033	.037	−.029	.971	.953–989	.002	.426
	Neck Disability Index (0–100%)	168	−.039	.962	.938–.986	.002	.080	−.061	.941	.909–.974	.001	.467
	Pain Disability Index (0–70)	170	−.027	.974	.948–.999	.044	.034	−.081	.922	.881–.965	.001	.474
Psychological	Pain Catastrophizing Scale (0–52)	166	−.033	.968	.934–1.003	.069	.028	−.030	.970	.926–1.016	.201	.357
	HAD Anxiety (0–21)	167	−.051	.950	.862–1.047	.301	.009	−.059	.943	.826–1.076	.382	.360
	HAD Depression (0–21)	166	−.023	.978	.889–1.076	.644	.002	−.100	.905	.789–1.037	.151	.390
	Self-Efficacy Scale (0–200)	164	−.004	.996	.986–1.005	.492	.007	−.011	.989	.975–1.004	.142	.399
	Tampa Scale of Kinesiophobia (11–44)	165	−.038	.963	.908–1.020	.197	.014	−.031	.969	.897–1.047	.430	.374
Musculoskeletal physical	NME, flexion (seconds)	159	−.012	.988	.978–.998	.014	.059	−.021	.980	.965–.994	.006	.439
	NME, extension (seconds)	154	−.001	.999	.997–1.001	.207	.016	.000	1.000	.998–1.001	.596	.402
	ROM, flexion (degrees)	165	.009	1.009	.984–1.035	.476	.004	−.010	.990	.955–1.027	.601	.389
	ROM, extension (degrees)	165	−.010	.990	.965–1.015	.418	.005	−.011	.989	.956–1.023	.512	.394
	ROM, flexion + extension (degrees)	165	.000	1.000	.983–1.016	.953	.000	−.005	.995	.973–1.017	.630	.399
	ROM, rotation right + left (degrees)	165	−.018	.982	.967–.998	.028	.042	−.023	.978	.955–1.001	.056	.402
Quality of life	EQ-5D Index (−.594–1.000)	165	−.631	.532	.148–1.915	.334	.008	−1.058	.347	.045–2.655	.308	.390
	EQ-VAS (0–100 mm)	166	−.007	.993	.978–1.003	.354	.007	−.025	.975	.953–.998	.030	.426

^a^ Adjusted for baseline measures; sex, age, WAD-level, Neck-Specific Exercise, Dizziness, and each separate predictor

Dependent variable, dizziness at 12 months; *VAS* visual analogue scale; *NME* Neck muscle endurance; *ROM* range of motion, *HAD-A* HAD-D hospital anxiety and depression scales, *EQ-5D* EQ-VAS- EuroQuol quality of life index

Table 4 depicts the results of the final multivariable logistic regression model. Measures of change at 12 months in, NDI (OR .95, 95% CI .91–.98), and NME flexors (OR .99, 95% CI .97–1.00), showed significant association with the presence of dizziness at 12 months, based on the adjusted models. Indicating that the two predictors and the baseline covariates explain 50% of the variation in dizziness at 12 months. Noticeable is that the baseline covariates (predominantly age, dizziness and NDI at baseline) explain approximately 37% of the variation in dizziness at 12 months. The type of exercise intervention was not associated with the presence of dizziness 12 months post.

**Table 4:** Results of the final multiple logistic regression model on dependent variable dizziness at 12 months, using backward stepwise procedure- Nagelkerke pseudo R^2^ was 0.5 in the final model

Type of measures		B	Odds Ratio	95% CI for Odds Ratio	*p*-value	Pseudo R^2^
Predictors, measured as change score at 12 months	Constant	−4.356	.013		.001	.499
	Neck Disability Index (0–100%)	−.056	.946	.910–.983	.005	
	NME, flexion (seconds)	−.013	.987	.972–1.002	.085	
Covariates at baseline	Female	−.375	.687	.240–1.965	.687	
	Age	.050	1.051	1.009–1.096	.016	
	WAD-level 3	.550	1.734	.707–4.251	.229	
	Neck-specific exercise	.262	1.299	.461–3.663	.620	
	Dizziness	2.255	9.532	3.133–29.001	<.001	
	Neck Disability Index (0–100%)	.058	1.059	1.014–1.107	.010	
	NME, flexion (seconds)	−.003	.997	.984–1.009	.612	

Dependent variable, dizziness at 12 months; *NME* Neck muscle endurance

## Discussion

The aim of this study was to first compare cervical related physical and psychological factors in individuals with and without dizziness, 12 months after commencement of a neck specific (with or without a behavioural approach) or general exercise intervention, and secondly to determine the combination of these factors to best predict those reporting ongoing dizziness. The results demonstrated that those who complain of dizziness in the long term, overall have significantly higher levels of pain and disability and poorer neck physical and psychological function, quality of life and static balance at baseline and at 12 months post commencement of an exercise program compared to those not reporting these symptoms. Of these, the factors that best determined ongoing dizziness were age, baseline levels of dizziness and neck disability as well as less improvement in neck pain and disability and neck flexor muscle endurance at 12 months. The results of the study also confirmed that the symptom of dizziness is common in those with persistent WAD and was present in many participants, 12 months later despite some performing neck specific exercises previously shown to be favourable over general exercise for improving dizziness [12]. Overall these findings may have implications for future directions for management of persistent WAD.

### Dizziness

The symptom of dizziness was frequent in those with chronic WAD before exercise intervention (73%) and at a 12-month follow-up (63%). Similar to those reported by Treleaven et al. [4]. Further, although the levels of dizziness were not marked and as high as people with diagnosed vestibular pathology such as Menieres disease [27], they are likely clinically relevant as they were on average at a higher level than those seen in people, for example, 6 months after acute vestibular loss [28] and similar to those with symptoms several years after acute vestibular loss [29]. This would suggest that the symptom of dizziness is clinically relevant and should be considered in those with persistent WAD and assessment and management specifically directed towards this. Interestingly 6% of the total cohort not reporting dizziness at baseline reported some dizziness at the 12-month follow up (Fig. 1). This may reflect the known yearly point prevalence of dizziness and the possibility of onset of dizziness from other causes [30]. Future work could consider the longterm epidemiology of dizziness post whiplash.

### The final multivariable model; NDI and NME flexors

Although the change in scores from baseline to the 12-month post intervention follow-up in neck pain intensity, NDI, PDI, EQ-VAS and NME flexors were significant factors in the logistic regression, NDI and NME flexors were the remaining variables in the final model and together with age, baseline dizziness and NDI, explained 50% of the variance. These results strengthen the possibility of the role of disturbed cervical afferent input contributing to the cause of dizziness in some of the present population. This could mean that efforts directed towards reducing neck pain and disability and to exercise the cervical flexors may be important to reduce dizziness. Alternatively, this could suggest that the presence of dizziness inhibits the success of such interventions.

Interestingly neck extensor muscle endurance or muscle fatigability has been associated with greater balance deficits in WAD [7] which is often related with the symptom of dizziness [6]. In the current study though, neck flexors, not extensors, was a predictor for long term dizziness. Although, this may have been due to the method of testing with the extensor clinical test being highly variable with some people reaching long holding times [31]. In individuals with chronic WAD altered neck muscle interaction patterns of lower activity of the deep and an increased activity in the superficial neck muscles [32], and elongation of the deep neck muscles and a more stereotypic ventral movement pattern compared with healthy individuals has been identified [33]. Supporting the findings, neck-specific exercises have shown to improve ventral neck muscle interaction [34] and reduce dizziness, headache and health-related quality of life in chronic WAD and to be superior compared with general physical activity [12].

Alternatively, the results could indicate that the presence of dizziness for most may be a factor inhibiting response to exercise programs aimed at reducing neck pain and disability and improving neck muscle function. In the current study, the dizzy group only improved by about 1 second compared to 17 seconds in the non-dizzy group in flexion endurance and similarly no clinically relevant change in NDI (2%) was seen in the dizzy group compared to about 9% in the non-dizzy group. Similar findings with respect to the presence of dizziness and effectiveness of cervical management has been seen in those with cervicogenic headache [35]. A previous study also demonstrated a mild to moderate relationship between a change in dizziness intensity and NDI [12]. In this case, treatment directed towards dizziness would seem appropriate to assist recovery. Interestingly an oculomotor rehabilitation program demonstrated improvements in balance and symptoms of dizziness in patients with chronic WAD but not neck pain intensity [36, 37]. Perhaps a multimodal approach with specific tailored sensorimotor control exercises in conjunction with local treatment directed towards neck specific exercises and improving neck pain will be required.

### Measures of sensorimotor control

The results also suggest balance deficits remain regardless of symptoms of dizziness. The average sharpened Romberg score was lower in the dizzy compared to non-dizzy group. However, values for both groups suggest many participants may have deficits in this static balance test. Further, as balance is known to decline in association with vestibular and visual changes with ageing [38] it may be important to specifically assess and address this for falls prevention in this group, however, more research is required.

Head repositioning accuracy which is thought to be a measure of cervical proprioception, was not significantly higher in the dizzy group and on average was within normal limits, however, 46% of patients still had an abnormal score > 4.5 degrees in at least one direction of movement, which also supports a possible role of cervical proprioception as a contributor to dizziness in this population [39].

### Range of motion

Those complaining of dizziness also had less total neck range of motion (*p* = 0.001) than those not complaining of these symptoms and especially regarding change in the horizontal planes (R^2^ 0.042). It is possible that this may be related to the presence of dizziness with movement or fear of motion [40] although scores of fear of movement were relatively low in both groups.

### Psychological factors

Higher scores on a depression scale and poorer quality of life were identified in the participants with dizziness. Interestingly all of 12 participants identified as at risk of probable depression HAD D (> 10) were in the dizzy group, although this only accounted for 13% of the participants in the dizzy group, suggesting levels of depressive symptoms are not generally high in those with WAD. Interestingly though, depression has been associated with persistent symptoms such as dizziness following acute vestibular loss [28]. Further, although, anxiety is usually associated with dizziness, [41] this not found in the current study. Whilst it is difficult to determine whether or not the psychological factors caused or worsened or were induced by the dizziness, high pain levels and dizziness have been previously identified as factors associated with initial and persistent depression in those with WAD [42]. The results of this study would concur with this finding.

Quality of life ratings were low (0.61) in the dizzy group after rehabilitation and similar to those post-surgery for cervical disc disease [43] and low compared to other illnesses such as (0.79) in patients with asthma [44]. This would indicate that more is to be done to improve quality of life and burden of the disorder in this group.

Mean results for pain catastrophising (PCS) and fear of movement (TSK) were significantly poorer in the dizzy group but the mean scores for these questionnaires were low and likely not clinically relevant [45]. Similarly, although self-efficacy was significantly lower, mean values suggest this was not a main factor in this group.

### Strengths and limitations

The current study used a strong design, had a long-term follow-up period and has large subject numbers in each group to allow for exploration of factors associated with dizziness 12 months post an intervention in those with WAD. However, the study can only assess relationships and the precise reasons for dizziness at 12-month follow-up cannot be ascertained. Regardless, certain factors (NDI and NME flexion changes) were associated with dizziness at follow-up in association with baseline variables of age, dizziness, or NDI. This may infer a possible cervical role in the presentation of dizziness but cannot determine the cause or causes of dizziness. Interestingly whether the group performed neck specific exercises or not was not a factor. This suggests further exploration is needed. Other measures that could be related to dizziness, such as overall health, and emergence or contribution of other causes of dizziness, such as vestibular pathology, should also be considered in future research.

## Conclusion

The results of the study show that 63% of participants with persistent WAD had symptoms of dizziness, unsteadiness and deficits in balance and cervical proprioception, even 12 months post a specific neck- or general exercise program. This might be related to levels of change of NDI and NME flexion as well as baseline covariates. Pain and EQ-VAS were also factors of importance although not appearing in the final model. Alternatively, it may be that dizziness could be contributing to some of these signs and symptoms or have another cause. The results indicate a cervicogenic role in the production of dizziness in some and that intervention and rehabilitation specifically addressing neck-specific disability and NME flexion seem important. However, future directions should consider neck specific exercise with a multimodal approach, including tailored sensorimotor control exercises, as dizziness may be a factor inhibiting recovery in some individuals with persistent WAD. Future research should also explore other possible causes of dizziness in this cohort to assist management and reduce the ongoing burden and effects on quality of life.

## Data Availability

The datasets used and/or analysed during the current study are available from the corresponding author on reasonable request.

## Abbreviations

WAD: Whiplash associated disorder
NDI: Neck disability index
NME: Neck muscle endurance
VAS: Visual analogue scale
UCLA: University of California Los Angeles dizziness score
RCT: Randomised controlled trial
OR: Odds ratios
CI: 95% confidence intervals
HRA: Head relocation accuracy
ROM: Range of motion
PDI: Pain disability index
PCS: Pain catastrophising scale
HAD: Hospital anxiety and depression
TSK: Tampa scale of kinesiophobia
SES: Self efficacy scale
EQ-5D: Quality of life
